# Agreement, correlation, and kinetics of the alveolar bone-loss measurement methodologies in a ligature-induced periodontitis animal model

**DOI:** 10.1590/1678-7757-2016-0517

**Published:** 2017

**Authors:** Paula Katherine Vargas-Sanchez, Marcella Goetz Moro, Fabio André dos Santos, Ana Lia Anbinder, Eliane Kreich, Renata Mendonça Moraes, Lauryellen Padilha, Caroline Kusiak, Dionizia Xavier Scomparin, Gilson Cesar Nobre Franco

**Affiliations:** 1 Universidade Estadual de Ponta Grossa, Departamento de Odontologia, Ponta Grossa, PR, Brasil; 2 Univ Estadual Paulista, Departamento de Biociências e Diagnóstico Oral, São José dos Campos, SP, Brasil; 3 Universidade Estadual de Ponta Grossa, Programa de Pós-Graduação em Ciências Biomédicas, Ponta Grossa, PR, Brasil; 4 Universidade Estadual de Ponta Grossa, Programa de Graduação em Enfermagem, Ponta Grossa, PR, Brasil; 5 Universidade Estadual de Ponta Grossa, Departamento de Biologia, Ponta Grossa, PR, Brasil

**Keywords:** Experimental animal models, Periodontitis, Alveolar bone loss

## Abstract

Periodontal research involves the use of animal models to better understand the biological processes of periodontal diseases and the potential of new or existing therapies. Currently, ligature-induced periodontitis in rats is the main model used in periodontal research, in this model, alveolar bone loss (ABL) is the main parameter evaluated by radiographic, morphometric, and histological techniques. Interestingly, although these methodologies are widely used, it is not totally clarified neither the kinetics of ABL over the induction time nor the agreement degree (repeatability and reproducibility) of these techniques. Objective: To characterize ABL kinetics at 0, 3, 7, 15, 30, and 60 days after ABL induction by ligature and to evaluate the intra- (repeatability) and inter-examiner (reproducibility) agreement and the correlation among the radiographic, morphometric, and histological methodologies. Material and Methods: 60 male Wistar rats with induced ABL were randomly divided into 6 experimental groups (n = 10 animals/group). After 0, 3, 7, 15, 30, and 60 days, the animals were euthanized and their hemimandibles were removed for ABL determination using radiographic, morphometric and histological techniques. Results: Radiographic and morphometric/linear techniques allowed the detection of statistically significant ABL on the third day, while histological and morphometric/area techniques could only detect ABL after the seventh day (ANOVA/Tukey, p<0.05). After the fifteenth day, except for histological analysis, the ABL was stabilized. Concerning the agreement of the methodologies, Bland Altman's test (intra and inter-examiner evaluations) showed no difference among the measurements (p>0.05). In addition, high correlations (Pearson's test, r2>0.9, p<0.05) were observed. Conclusion: The results indicated that the minimum time for ABL induction could vary from 3 to 7 days, according to the chosen analysis methodology. Agreement and correlation data support the comparison of results between studies with same induction time.

## Introduction

In periodontal research, animal models are important for studying the relationship between diseases and external factors, and also for testing potential new therapies. The model selection will depend on the aim of the study, but in general, an adequate model should present biological events similar to that observed in humans, such as the progressive destruction of connective tissue, bone loss, and the formation of periodontal pockets^2^.

The rat is the most widely used rodent to study the pathogenesis of periodontal disease (PD) because of the similarity between the tissue structure of the gingival area and the bone/collagen degradation observed during alveolar bone loss (ABL) in rats and humans^19^. The main models used to induce ABL are: 1- Ligature-induced periodontitis: the insertion of yarn around the cervix of a mandibular molar; 2- LPS-induced periodontitis: the application of lipopolysaccharide (LPS) into mandibular gingiva; and 3- Microorganism-induced periodontitis: the inoculation of specific periodontal pathogens^1^ ^,^ ^5^ ^,^ ^20^.

Regardless of the periodontitis induction method chosen, radiographic, morphometric and histological techniques are the most cited techniques in the literature used to quantify ABL, which is the main parameter analyzed for this animal model. The radiographic method usually involves analyzing ABL by measuring the linear distance between the cementoenamel junction (CEJ) in the alveolar bone crest of the first mandibular molar^8^ ^,^ ^9^ ^,^ ^13^. The morphometric method requires measuring the linear distance (mm) or the area (mm^2^) between the CEJ and the bone crest in the mandibular molars^3^ ^,^ ^14^. In the histological method, the interproximal bone loss between the first and second molars can be evaluated by the linear distance between the CEJ and the bone crest^18^.

Considering the relevance of ABL parameter for periodontal research, the establishment of the reliability, agreement (repeatability and reproducibility) and correlation of their main analysis methods is one of the most important principles in science, since it allows the data to be interchangeable among studies carried out with similar proposals. In addition, the knowledge the kinetics of alveolar bone loss over time of the methodologies is also considered essential to the success of a study (study design and interpretation of results) because each technique evaluates a specific region in the periodontal tissue and consequently it becomes possible the occurrence of different rates of alveolar bone degradation according to the ABL model and the induction time chosen. Nowadays, the time of ABL induction in this animal model is found in the literature ranging from 1 to 60 days^9^ ^,^ ^10^ ^,^ ^12^ ^,^ ^13^.

Therefore, the aim of this study was to determine the kinetics of alveolar bone loss 0, 3, 7, 15, 30, and 60 days after ligature-induced periodontitis in rats and to assess the Bland-Altman's agreement and Pearson's correlation of radiographic, morphometric, and histological methodologies for ABL analysis.

## Material and Methods

The experimental protocol followed the ARRIVE guidelines for animal research suggested by the National Center for the Replacement, Refinement, and Reduction for Animals in Research^11^. The sample size was calculated with the software G*Power 3.1^6^. Assuming a significant difference greater than 1.5 times the standard deviation in the ABL parameter and a Type I (α) and Type II (β) errors of 5% and 20%, respectively, the sample size was estimated in nine animals (minimum) *per* group.

Sixty male Wistar rats weighing approximately 250 g each were included in the study. The animals were maintained in plastic cages (5 animals/cage) under a 12-hour light/dark cycle at a temperature of 22°C, and received food and water *ad libitum.* The study was approved by the Ethics Committee of Animal Use (CEUA – Protocol: 014/2013).

The animals were randomly divided into six experimental groups (n = 10 animals/group) according to the time of periodontitis induction (G0: 0, G3: 3, G7:7, G15: 15, G30: 30, and G60: 60 days) by ligature method.

### Periodontitis induction

**Figure 1:**
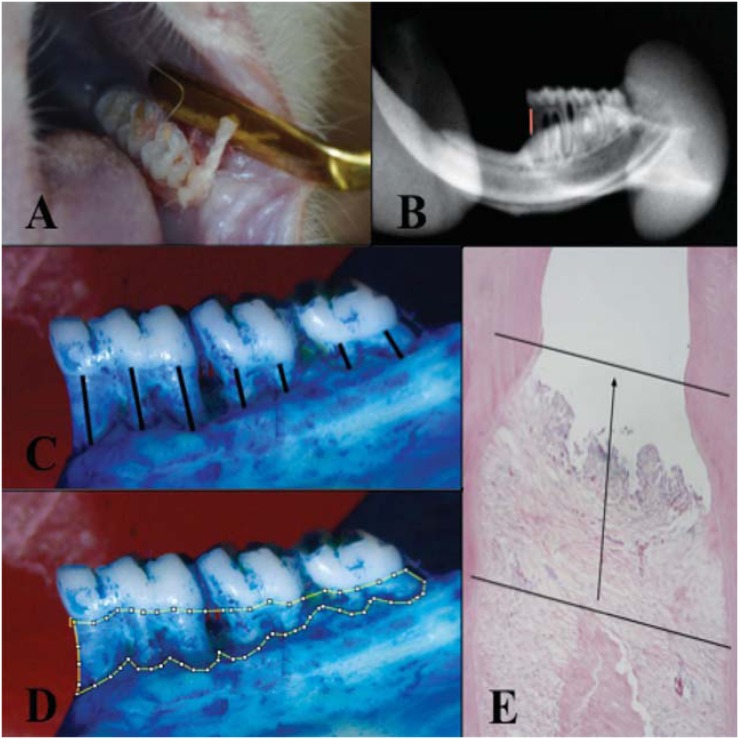
(A) Ligature-induced periodontitis methodology; (B) parameters used for radiographic measurement (mm); (C) parameters used for morphometric (linear, mm) measurement; (D) parameters used for morphometric (area, mm^2^) measurement; and (E) parameters used for histological measurement (mm)

Under general anesthesia (IP, ketamine-10%, 90 mg/kg, and xylazine-2%, 10 mg/kg), a cotton ligature (Coats Corrente, São Paulo, SP, Brazil) was inserted around the cervix of the first mandibular molars (Figure 1A). After the experimental periods (0, 3, 7, 15, 30, or 60 days post-periodontitis induction), the animals were sacrificed by anesthetic overdose and their hemimandibles were excised and fixed in 10% formalin solution for 48 hours. The right side was used for morphometric and radiographic analysis, while the left side was used for histological analysis.

### Radiographic analysis

The hemimandibles were radiographed using a digital x-ray system (Kavo Dental, Biberach, Baden-Wurttemberg, Germany). The x-ray incidence was lingual, with an exposure time of 0.04 seconds and an object-focus distance of 30 cm. The mesial linear distance (mm) between the CEJ and the alveolar bone crest of the first molar was determined using the software Image J 1:48 (Figure 1B).

### Morphometric analysis

The hemimandibles were boiled for 30 minutes and immersed in hydrogen peroxide solution (3%) to remove the excess tissue. After this procedure, the hemimandibles were stained with methylene blue 1% to demarcate the CEJ. For each specimen, the following were measured: 1) linear distance (mm) between the CEJ and the bone crest of the three mandibular molars, with a total of seven measurements corresponding to the number of roots, and 2) the area (mm^2^) of bone loss between the CEJ and the bone crest of the three molars. The measurements were made on the lingual surface using the software Image J 1.48 (Figure 1C/D).

### Histological analysis

The hemimandibles were decalcified with EDTA 4% and stained with hematoxylin eosin (HE). Measurements of the linear distance (mm) between the CEJ and the interproximal alveolar crest, the first molar, and the second molar were obtained. The measurements were made using the software Image J 1.48 (Figure 1E).

### Data evaluation and statistical analysis

Intra-examiner agreement (repeatability) of the ABL measurement techniques:

A trained examiner performed two independent ABL measurements (radiographic, morphometric, and histological techniques) using the G15 group with a two-week interval. Bland-Altman's test followed by a *t* test (one sample) was carried out to determine the intra-examiner agreement.

Inter-examiner agreement (reproducibility) of the ABL measurement techniques:

Two trained examiners performed independent ABL measurements (radiographic, morphometric, and histological techniques) using the G15 group. Bland-Altman's test followed by a *t* test (one sample) was carried out to determine the inter-examiner agreement.

Correlation among the ABL measurement techniques:

Using data from G15 group, Pearson's correlation was carried out to determine the correlation among the different methodologies of ABL evaluation.

Kinetics of bone loss observed in the ABL measurement techniques:

ANOVA and Tukey's test were used to determine the statistical differences in ABL among the periods of evaluation with the software Graph Pad Prism 6.0e.

The level of significance was 5% in all tests.

## Results

### ABL Kinetics

**Figure 2:**
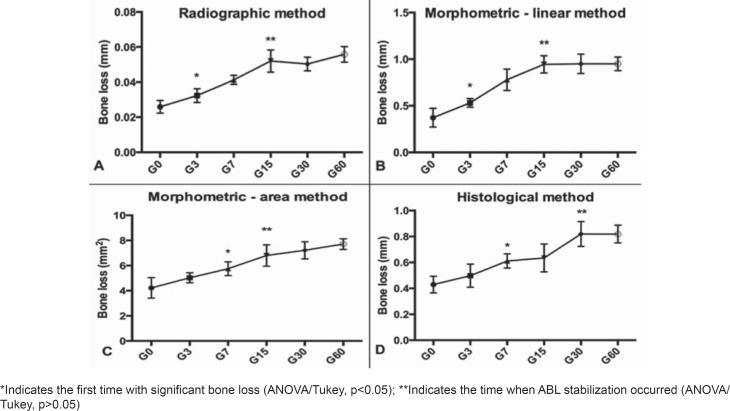
Bone loss for each methodology in relation to the period of PD induction using the (A) radiographic method; (B) morphometric/linear method; (C) morphometric/area method; and (D) histological method

Both the radiographic and morphometric/linear analyses revealed significant bone loss on day 3. It was progressive with a statistical significance until day 15. After this period, the ABL remained constant until day 60 (Figure 2A and B).

Using morphometric/area analyses, after ABL induction, the minimum time needed to detect significant bone loss was 7 days. Afterward, progressive bone loss occurred until day 15, and from day 15 to day 60, the ABL remained constant (Figure 2C).

**Figure 3:**
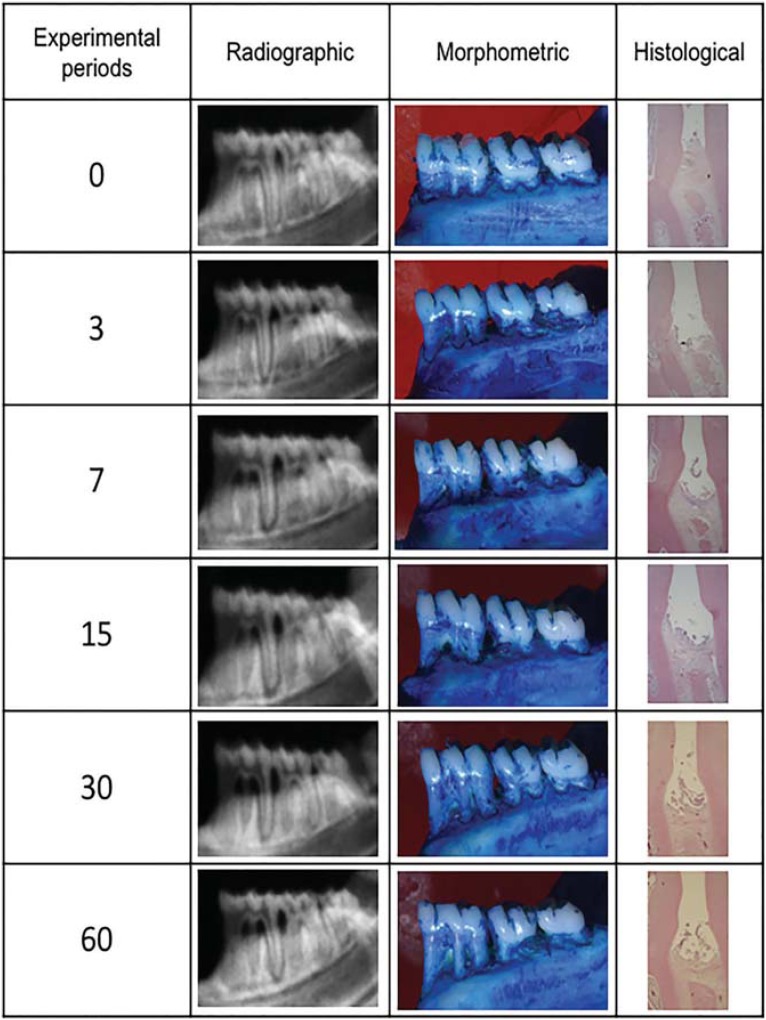
Analysis methodology (radiographic, morphometric and histologic) in different induction times

Using histological analyses, linear analysis showed significant bone loss at day 7, which was progressive until day 30. After this time, the bone resorption was uniform (Figure 2D). Figure 3 shows images of each analysis methodology (Radiographic, Morphometric and Histologic) in the different induction times.

### Agreement analysis

**Figure 4:**
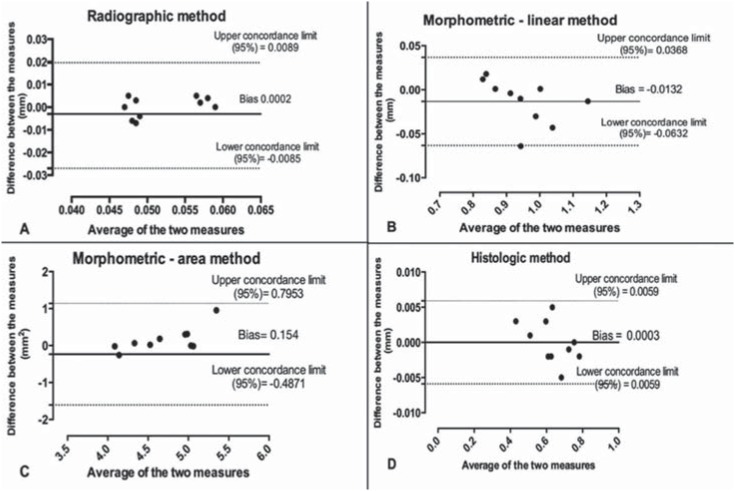
Bland–Altman's test (intra-examiner evaluation): (A) radiographic method; (B) morphometric/linear method; (C) morphometric/area method; and (D) histological method

**Figure 5:**
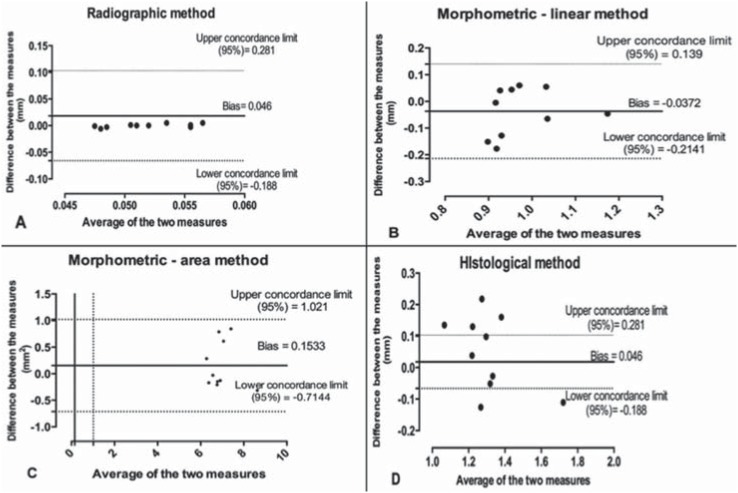
Bland–Altman's test (inter-examiner evaluation): (A) radiographic method; (B) morphometric/linear method; (C) morphometric/area method; and (D) histological method

Bland-Altman's test did not show any statistical differences in either the intra- or the inter-examiner for all evaluations (*p*>0.05) (Figure 4 and 5).

### Correlation analysis

Pearson's test indicated high correlations (*r* ^2^>0.9, *p*<0.05) between the ABL measurement methodologies evaluated (Table 1).

**Table 1:** Pearson's correlation (*r* ^2^) between the methodologies

	Radiographic	Morphometric/linear	Morphometric/area	Histological
Radiographic	–	0.98	0.99	0.91
Morphometric/linear	0.98	–	0.96	0.90
Morphometric/area	0.99	0.96	–	0.96
Histological	0.91	0.90	0.96	–

## Discussion

The use of animal models in periodontal research is becoming increasingly frequent. Between 1966 and 2016, more than 300 articles using experimental periodontitis induced by ligature in rats were published in the PubMed database that aimed to clarify biological processes or to search for new potential therapies related to PD. Although the ligature-induced periodontitis model has been described since the late 1960s^17^, interestingly, studies that characterize this model (ABL over the induction time), as well as the agreement (intra- and inter-examiner) and correlation degree of the main methodologies employed for ABL evaluation, are scarce. Taken together, these parameters allow for better design of the experimental protocol and support the comparison of results obtained by the same or different research groups.

The principle of induction of Periodontal Disease with the use of ligature is based not only on mechanical trauma, but mainly due to plaque accumulation, thus promoting a local immune-inflammatory reaction responsible for tissue inflammation and alveolar bone loss^17^. The dental biofilm in the ligature is similar to that observed in human disease, composed by *Actinomyces-, Fusobacterium-, Prevotella nigrescens-, Parvimonas micra-, Porphyromonas gingivalis-*and *Aggregatibacter actinomycetemcomitans-like* species^4^. The induction of periodontal disease in rats using ligature resembles human periodontitis, since the destructive phase is represented by the formation of an inflammatory infiltrate in the gingival tissue, which predates bone resorption^7^. In our study, we could observe this characteristic, since in GO group, inflammation was almost absent, while in the other groups, it was possible to observe a mild chronic inflammation, mainly composed by lymphocytes predominantly found around junctional epithelium, without significant differences among the experimental periods analyzed (G3, G7, G15, G30 and G60). The pattern of cell infiltration differs from human periodontal disease, where neutrophils are found in initial and early lesions, and plasma cells are predominant in established gingivitis and in periodontitis^15^.

In our study, ABL increased significantly, compared with the control, on the third day after PD induction, when measured by radiographic or morphometric/linear techniques, while ABL was detected after 7 days using morphometric/area and histological techniques. The rapid bone resorption described for this rat model and confirmed in our study may be due to the proximity of the ligature to the bone crest.

This knowledge supports the elaboration of short protocols for ABL evaluations, mainly in cases when is necessary to evaluate biological parameters associated with the triggering of inflammatory processes, including the initial mechanisms of bone resorption. For example, Rodini, et al.^16^ (2008) showed the iNOs gene has higher expression on the third day postperiodontitis induction when using ligature method, while higher expression for the MMP-9 gene occurred on the seventh day post-induction. In addition, after 7 and 15 days, no differences were detected in the gene expression of iNOs and MMP-9, respectively, compared with the control group. Based on this information, the appropriate choice of the induction time associated with the ABL measurement methodology represents key steps for a study's success^16^.

Additionally, ABL stabilized on the fifteenth day for all the methodologies but histological analysis, suggesting a chronic process after this period. Therefore, the study design should be adjusted according to the main objective and the results of studies performed with different induction times should be compared with caution. However, the correlation test suggests that a comparison could be carried out when considering the same induction time, even if different ABL measurement methodologies have been used.

In science, the basic principle of any methodology is that it is repeatable and reproducible. Every study should be reproducible by the same or different research groups, at any time. In this context, the Bland-Altman test results demonstrated excellent agreement (both in repeatability and reproducibility) among the methodologies evaluated, showing the reliability of the ABL measurement methodologies used in periodontal research involving animal models.

## Conclusions

The authors believe this is the first study exclusively designed to evaluate the kinetics, agreement and correlation of alveolar bone loss (ABL) among four different analysis methodologies. Therefore, the data of this study show that choosing the correct ABL measurement methodology is a crucial step during a study design, since the initial time of bone loss and the moment when this process stabilizes can vary according to the methodology chosen for this analysis. The agreement and correlation results support the comparison of results among studies with the same induction time, even when different ABL measurement methodologies have been used.
